# Bt Crops Producing Cry1Ac, Cry2Ab and Cry1F Do Not Harm the Green Lacewing, *Chrysoperla rufilabris*

**DOI:** 10.1371/journal.pone.0060125

**Published:** 2013-03-27

**Authors:** Jun-Ce Tian, Xiang-Ping Wang, Li-Ping Long, Jörg Romeis, Steven E. Naranjo, Richard L. Hellmich, Ping Wang, Elizabeth D. Earle, Anthony M. Shelton

**Affiliations:** 1 Department of Entomology, Cornell University/New York State Agricultural Experiment Station (NYSAES), Geneva, New York, United States of America; 2 State Key Laboratory Breeding Base for Zhejiang Sustainable Pest and Disease Control, Institute of Plant Protection and Microbiology, Zhejiang Academy of Agricultural Sciences, Hangzhou, China; 3 College of Agriculture, Yangtze University, Jingzhou, Hubei, China; 4 Rice Research Institute, Guangxi Academy of Agricultural Sciences, Nanning, Guangxi, China; 5 Agroscope Reckenholz-Tänikon Research Station ART, Zürich, Switzerland; 6 USDA-ARS, Arid Land Agricultural Research Center, Maricopa, Arizona, United States of America; 7 USDA–ARS, Corn Insects and Crop Genetics Research Unit and Department of Entomology, Iowa State University, Ames, Iowa, United States of America; 8 Department of Plant Breeding and Genetics, Cornell University, Ithaca, New York, United States of America; University of Tennessee, United States of America

## Abstract

The biological control function provided by natural enemies is regarded as a protection goal that should not be harmed by the application of any new pest management tool. Plants producing Cry proteins from the bacterium, *Bacillus thuringiensis* (Bt), have become a major tactic for controlling pest Lepidoptera on cotton and maize and risk assessment studies are needed to ensure they do not harm important natural enemies. However, using Cry protein susceptible hosts as prey often compromises such studies. To avoid this problem we utilized pest Lepidoptera, cabbage looper (*Trichoplusia ni*) and fall armyworm (*Spodoptera frugiperda*), that were resistant to Cry1Ac produced in Bt broccoli (*T. ni*), Cry1Ac/Cry2Ab produced in Bt cotton (*T. ni*), and Cry1F produced in Bt maize (*S. frugiperda*). Larvae of these species were fed Bt plants or non-Bt plants and then exposed to predaceous larvae of the green lacewing *Chrysoperla rufilabris*. Fitness parameters (larval survival, development time, fecundity and egg hatch) of *C. rufilabris* were assessed over two generations. There were no differences in any of the fitness parameters regardless if *C. rufilabris* consumed prey (*T. ni* or *S. frugiperda*) that had consumed Bt or non-Bt plants. Additional studies confirmed that the prey contained bioactive Cry proteins when they were consumed by the predator. These studies confirm that Cry1Ac, Cry2Ab and Cry1F do not pose a hazard to the important predator *C. rufilabris*. This study also demonstrates the power of using resistant hosts when assessing the risk of genetically modified plants on non-target organisms.

## Introduction

Green lacewings (Neuroptera: Chrysopidae) are important beneficial predators in many cropping systems [1]. The biological control function provided by lacewings and other natural enemies is regarded as a protection goal that should not be harmed by the application of any new pest management tool [2], [3]. Consequently, the impact of insect-resistant genetically engineered (GE) plants that produce Cry proteins derived from the bacterium *Bacillus thuringiensis* on valued non-target arthropods should be addressed in the ecological risk assessment that precedes the commercial release of any new GE plant.

The initial steps in the risk assessment are laboratory studies that provide information on whether the insecticidal protein is toxic to selected surrogate test species under worst-case exposure conditions [4], [5]. Such laboratory studies need to be carefully designed to provide robust data that can be interpreted and thus support the ecological risk assessment [6]. One key element of such studies is to ensure that the test insects are exposed to high doses of a biologically active Cry protein. This can be achieved in several ways. First, the test protein can be incorporated in an artificial diet. Second, the test substance can be mixed with non-GE plant material or provided in the form of GE plant material. Third, predatory species such as lacewing larvae can be exposed to the plant-produced Cry proteins through GE-plant fed herbivores that are used as prey. While the latter case has the advantage of providing a very realistic exposure pathway, it carries the risk that the herbivores themselves are affected by the test substance and consequently effects seen on the predator may be due to a lower prey quality rather than a direct effect of the plant-produced Cry protein. Such so-called ‘prey-quality mediated effects’ have been observed in numerous tri-trophic feeding studies with Bt-transgenic crops [7], [8] and have erroneously been interpreted as direct toxic effects of the Cry proteins [9], [10], [11], [12]. One way to avoid the impact of prey-quality mediated effects is to use herbivores as toxin carriers that are not susceptible to the plant expressed Cry proteins or strains of susceptible species that are highly resistant to the particular test compound [6]. Resistant strains of Lepidoptera have been used to assess the impact of particular Cry proteins in Bt plants on several natural enemies, including *Chrysoperla carnea* (Neuroptera: Chrysopidae) [13], *Coleomegilla maculata* (Coleoptera: Coccinellidae) [14], [15], *Pterostichus madidus* (Coleoptera: Carabidae) [16], and the parasitoids *Diadegma insulare* (Hymenoptera: Ichneumonidae) [17] and *Cotesia plutellae* (Hymenoptera: Braconidae) [18].

In the present study, we expand on the previous work on lacewings [13] by using different hosts, multiple toxins and several Bt plant species. Specifically, we use two different Bt-resistant Lepidoptera species, cabbage looper (*Trichoplusia ni*, Noctuidae) and fall armyworm (*Spodoptera frugiperda*, Noctuidae), to assess the direct toxic effects of Cry1Ac produced in Bt broccoli, Cry1Ac/Cry2Ab produced in Bt cotton, and Cry1F produced in Bt maize on larvae of the green lacewing, *Chrysoperla rufilabris* (Neuroptera: Chrysopidae). We selected this test species since it is a common predator in different crops including cotton [19] and is used in augmentative biological control programs [20] and is commercially available [21].

## Results

### Bt Proteins Levels in Bt Crops, Prey and Predators

Cry1Ac broccoli contained a mean of 10.15 µg Cry1Ac/g fresh weight (FW). The average Cry1Ac level in *T. ni* that fed on Cry1Ac broccoli was 3.5-fold lower than the Cry1Ac level in leaves, and was 22-fold higher than the Cry1Ac level in *C. rufilabris* that had fed on Cry1Ac broccoli-fed *T. ni* (Table 1). The differences were highly significant (*F* = 67.81; *df* = 2,8; *P*<0.001).

**Table 1:** Bt protein levels in Bt crops (broccoli, cotton and maize), prey (*Trichoplusia ni* and *Spodoptera frugiperda*) and the predator (3rd instar *Chrysoperla rufilabris*).

Sample	Measurement unit	Broccoli	Cotton		Maize
		Cry1Ac	Cry1Ac	Cry2Ab	Cry1F
Leaves	µg/g FW	10.15±1.20 a	1.15±0.17 a	29.51±0.38 a	2.72±0.06 a
Prey	µg/g FW	2.87±0.75 b	0.055±0.01 b	1.15±0.13 b	0.128±0.01 b
*C. rufilabris*	ng/g FW	129.10±37.37 c	7.88±1.46 c	51.55±7.4 c	14.22±5.70 c

Means (± SE) within a column followed by different letters are significantly different (One-way ANOVA, *P*<0.05); N = 3.

Prey: *T. ni* for broccoli and cotton, *S. frugiperda* for maize. FW: Fresh weight. Note that unit for leaves and prey is µg/g FW and for *C. rufliabris* ng/g FW.

The average Cry1Ac concentration in Bt cotton used in this study was 1.15 µg/g FW and the average Cry2Ab concentration was 29.51 µg/g FW. The average Bt protein concentration in *T. ni* that had fed on Bt cotton was 21-fold lower for Cry1Ac and 26-fold lower for Cry2Ab compared to the concentration in Bt cotton leaves. Furthermore, the Bt protein level in *C. rufilabris* was 7-fold lower for Cry1Ac and 22-fold lower for Cry2Ab than levels in *T. ni*. All differences were highly significant (For Cry1Ac: *F* = 230.3; *df* = 2,8; *P*<0.001; For Cry2Ab: *F* = 870.9; *df* = 2,8; *P*<0.001).

Cry1F maize leaves expressing approximately 2.72 µg/g FW were used in this study. *S. frugiperda* feeding on Cry1F maize contained 21-fold lower levels of Cry1F than maize leaves. The average Cry1F protein level in *C. rufilabris* that had fed on Cry1F maize-fed *S. frugiperda* was 9-fold lower than those in *S. frugiperda*. The Cry1F protein levels among plant, prey and predator were significantly different (*F* = 64.98; *df* = 2,8; *P*<0.001).

As expected, no Bt proteins were detected in non-Bt plants, prey fed non-Bt plants or predators fed prey on non-Bt plants.

### Prey-mediated Effects of Cry1Ac Broccoli on *C.* *rufilabris*

Ninety newly hatched *C. rufilabris* were provided Bt-susceptible *T. ni* that were fed non-Bt broccoli, Cry1Ac-resistant *T.ni* fed non-Bt broccoli, or Cry1Ac-resistant *T. ni* fed Cry1Ac broccoli (30 replications for each treatment). The different prey provided did not have an effect on the life-table parameters, including larval or pupal development time, fecundity or egg hatching rate of *C.* *rufilabris* (Table 2). Similar results were found when the lacewings were tested for a second generation (Table 2).

**Table 2:** Tri-trophic effects on life table parameters (means ± SE) of *Chrysoperla rufilabris* when fed *Trichoplusia ni* larvae that were reared on Cry1Ac-producing broccoli leaves or non-Bt broccoli leaves over two generations.

Parameters	Non-Bt broccoli Susceptible *T. ni*	Non-Bt broccoliResistant *T. ni*	Cry1Ac broccoliResistant *T. ni*	
1^st^ Generation				
*Survival (%)	83.3	80.0	83.3	χ^2^ = 0.17; df = 2; P = 0.92
†Larval stage (days)	9.9±0.1 (25)	9.7±0.1 (25)	9.6±0.1 (27)	*F* = 2.10 *df* = 2, 76; *P* = 0.13
†Pupal stage (days)	9.4±0.1 (25)	9.2±0.1 (24)	9.3±0.1 (25)	*F* = 0.94; *df* = 2, 73; *P* = 0.40
†Larva to adult (days)	19.8±0.2 (25)	18.9±0.1 (24)	18.8±0.1 (25)	*F* = 2.41; *df* = 2, 73; *P* = 0.10
†Total fecundity	217.4±11.9 (8)	236.6±22.9 (8)	233.1±15.5 (8)	*F* = 0.34; *df* = 2, 23; *P* = 0.71
†Egg hatching rate (%)	84.72±6.05 (3)	86.11±1.39 (3)	86.11±3.67 (3)	*F* = 0.04; *df* = 2, 8; *P* = 0.96
2^nd^ Generation				
*Survival (%)	80.0	76.7	80.0	χ^2^ = 0.14; df = 2; P = 0.93
†Larval stage (days)	10.6±0.2 (27)	10.4±0.2 (26)	10.4±0.1 (26)	*F* = 0.91; *df* = 2, 78; *P* = 0.41
†Pupal stage (days)	9.5±0.1 (24)	9.6±0.1 (23)	9.8±0.1 (24)	*F* = 2.80; *df* = 2, 70; *P* = 0.07
†Larva to adult (days)	20.2±0.2 (24)	20.0±0.1 (23)	20.0±0.1 (24)	*F* = 0.60; *df* = 2, 70; *P* = 0.55
†Total fecundity	214.9±23.8 (8)	228.0±22.5 (8)	218.4±12.1 (8)	*F* = 0.25; *df* = 2, 23; *P* = 0.78
†Egg hatching rate (%)	83.33±6.36 (3)	84.72±1.39 (3)	86.11±3.67 (3)	*F* = 0.10; *df* = 2, 8; *P* = 0.90

Number of replications is given in parenthesis. The experiment started with 30 larvae in each treatment.

* Wilcoxon test (*P*<0.05).

† One-way ANOVA (*P*<0.05).

### Prey-mediated Effects of Cry1Ac/Cry2Ab Cotton on *C.* *rufilabris*

Bt-susceptible *T. ni* were fed non-Bt cotton and Cry1Ac/Cry2Ab-resistant *T. ni* were fed non-Bt cotton or Cry1Ac/Cry2Ab cotton foliage, before being fed to newly hatched *C. rufilabris* (30 replications for each treatments). As in the previous experiments, there were no significant differences found for any of the life table parameters of *C. rufilabris* among the three treatments over two generations (Table 3).

**Table 3:** Tri-trophic effects on life table parameters (means ± SE) of *Chrysoperla rufilabris* when fed *Trichoplusia ni* larvae that were reared on Cry1Ac/Cry2Ab-producing cotton leaves or non-Bt isoline cotton leaves over two generations.

Parameters	Non-Bt cotton Susceptible *T. ni*	Non-Bt cottonResistant *T. ni*	Cry1Ac/Cry2Ab cottonResistant *T. ni*	
1^st^ Generation				
*Survival (%)	86.7	83.3	93.3	χ^2^ = 1.25; df = 2; *P* = 0.54
†Larval stage (days)	10.5±0.1 (27)	10.6±0.1 (26)	10.7±0.1 (28)	*F* = 0.88; *df* = 2, 80; *P* = 0.42
†Pupal stage (days)	9.6±0.4 (26)	9.2±0.1 (25)	9.4±0.1 (28)	*F* = 0.75; *df* = 2, 78; *P* = 0.48
†Larva to adult (days)	20.1±0.4 (26)	19.9±0.1 (25)	20.1±0.14 (28)	*F* = 0.33; *df* = 2, 78; *P* = 0.72
†Total fecundity	216.3±22.5 (8)	235.3±23.4 (8)	230.8±17.2 (8)	*F* = 0.22; *df* = 2, 23; *P* = 0.81
†Egg hatching rate (%)	81.94±5.01 (3)	81.94±0.01 (3)	83.33±0.02 (3)	*F* = 0.06; *df* = 2, 8; *P* = 0.93
2^nd^ Generation				
*Survival (%)	86.7	76.7	80.0	χ^2^ = 0.62; df = 2; *P* = 0.73
†Larval stage (days)	10.7±0.1 (27)	10.9±0.1 (26)	10.7±0.1 (25)	*F* = 0.41; *df* = 2, 77; *P* = 0.67
†Pupal stage (days)	9.7±0.4 (26)	9.7±0.1 (23)	9.7±0.1 (24)	*F* = 0.01; *df* = 2, 72; *P* = 0.99
†Larva to adult (days)	20.4±0.4 (26)	20.1±0.4 (23)	20.5±0.2 (24)	*F* = 0.43; *df* = 2, 72; *P* = 0.65
†Total fecundity	263.6±28.5 (8)	255.8±22.6 (8)	240.2±24.0 (8)	*F* = 0.17; *df* = 2, 23; *P* = 0.85
†Egg hatching rate (%)	80.56±3.67 (3)	81.94±3.67 (3)	83.33±4.17 (3)	*F* = 0.13; *df* = 2, 8; *P* = 0.88

Number of replications is given in parenthesis. The experiment started with 30 larvae in each treatment.

* Wilcoxon test (*P*<0.05).

† One-way ANOVA (*P*<0.05).

### Prey-mediated Effects of Cry1F Maize on *C. rufilabris*

Fifty newly hatched *C. rufilabris* were fed Cry1F-resistant *S. frugiperda* that fed on non-Bt maize. The same number of larvae was provided Cry1F-resistant *S. frugiperda* that had fed on Bt maize. Approximately 40% of the *C. rufilabris* reached the adult stage. No significant differences were detected for any life table parameters between the control (non-Bt) maize treatment and the Cry1F maize treatment in the first or second generations of *C. rufilabris* (Table 4).

**Table 4:** Tri-trophic effects on life table parameters (means ± SE) of *Chrysoperla rufilabris* when fed Cry1F-resistant *Spodoptera frugiperda* larvae that were reared on Cry1F-producing maize leaves or non-Bt maize leaves over two generations.

Parameters	Non-Bt maize	Cry1F maize	
1^st^ Generation			
*Survival (%)	40.0	42.0	χ^2^ = 1.57; df = 2; *P* = 0.21
†Larval stage (days)	14.2±0.2 (28)	14.0±0.2 (32)	*t* = 0.63; df = 58; *P* = 0.53
†Pupal stage(days)	10.2±0.1 (20)	10.3±0.1 (21)	*t* = 0.73; df = 39; *P* = 0.47
†Larva to adult (days)	24.2±0.2 (20)	24.1±0.3 (21)	*t* = 0.12; df = 39; *P* = 0.91
†Total fecundity	237.8±29.2 (8)	250.6±35.4 (8)	*t* = 0.28; df = 14; *P* = 0.78
†Egg hatchingrate (%)	81.94±3.67 (3)	84.72±2.78 (3)	*t* = 0.60; df = 4;*P* = 0.58
2^nd^ Generation			
*Survival (%)	36.0	44.0	χ^2^ = 1.05; df = 2; *P* = 0.30
†Larval stage (days)	14.1±0.3 (29)	14.3±0.2 (36)	*t* = 0.45; df = 63; *P* = 0.66
†Pupal stage(days)	10.0±0.2 (18)	9.7±0.1 (22)	*t* = 1.43; df = 38; *P* = 0.16
†Larva to adult(days)	23.8±0.2 (18)	23.8±0.2 (22)	*t* = 0.04; df = 38; *P* = 0.97
†Total fecundity	263.9±47.2 (8)	257.4±52.1 (8)	*t* = 0.09; df = 14; *P* = 0.93
†Egg hatchingrate (%)	84.72±5.01 (3)	83.33±2.41 (3)	*t* = 0.25; df = 4;*P* = 0.82

Number of replications is given in parenthesis. The experiment started with 50 larvae in both treatments.

* Wilcoxon test (*P*<0.05).

† Student’s *t*-test (*P*<0.05).

### Bioactivity of Bt Protein Residues after Ingestion by *T. ni* and *S. frugiperda*

In order to examine whether Bt proteins were still bioactive after ingestion by *T. ni* or *S. frugiperda*, Bt plant-fed and non-Bt plant-fed *T. ni* and *S. frugiperda* were collected. Samples were ground and diluted in PBST solution, and the solution was applied to cabbage leaf disks fed to Bt-susceptible diamondback moth, *Plutella xylostella* (Lepidopetra: Plutellidae), larvae. Extracts from Cry1Ac broccoli-fed *T. ni* larvae, Cry1Ac/Cry2Ab cotton-fed *T. ni* larvae and Cry1F maize-fed FAW larvae were toxic to Bt-susceptible *P. xylostella* larvae (*F* = 17.94; *df* = 6,34; *P*<0.001) (Table 5). This confirmed that the predator *C. rufilabris* was exposed to bioactive Bt proteins in all tri-trophic bioassays.

**Table 5:** Bioactivity to Bt-susceptible *Plutella xylostella* larvae to Bt proteins residues from *Trichoplusia ni* reared on Cry1Ac broccoli or on Cry1Ac/Cry2Ab cotton leaves and from *Spodoptera frugiperda* reared on Cry1F maize leaves**.**

Treatment	Mortality % (means ± SE)
*T. ni* reared on Cry1Ac broccoli leaf for 48 h	54.0±6.78 b
*T. ni* reared on non-Bt broccoli leaf for 48 h	12.0±3.74 a
*T. ni* reared on Cry1Ac/Cry2Ab cotton leaf for 48 h	44.0±8.94 b
*T. ni* reared on non-Bt cotton leaf for 48 h	8.0±5.83 a
*S. frugiperda* reared on Cry1F maize leaf for 48 h	70.0±7.75 b
*S. frugiperda* reared on non-Bt maize leaf for 48 h	8.0±3.74 a
dH_2_O (Control)	4.0±2.45 a

A total of 50 susceptible *P. xylostella* larvae were used in each treatment with 5 replications (10 larvae/replication). Mortality assessed after 72 h. Means followed by different letters are significantly different (One-way ANOVA, *P*<0.05).

## Discussion

The commercialization of plants producing insecticidal crystal (Cry) proteins from *Bacillus thuringiensis* (Bt) for insect management has revolutionized agriculture [22] and become a major tool for integrated pest management (IPM) programs [23]. In 2011, Bt crops (cotton and maize) were grown on more than 66 million ha in 26 countries [24]. Two major concerns about Bt plants have been their potential effects on non-target organisms, especially those natural enemies that help suppress pest populations [23], and the pest insect’s potential ability to evolve resistance to the Bt proteins [25]. Both areas are the focus of studies in many laboratories.

In maize, the fall armyworm, *S. frugiperda*, is a major pest in the Americas and was the first insect to have evolved resistance in the field to Cry1F to such an extent that it caused extensive damage to the crop and Bt maize and was removed from the market in Puerto Rico [26]. Cotton is one of the main hosts for *T. ni* [27] and infestations can result in yield loss of 30% to 92% [28]. A population of *T. ni* evolved resistance to a Bt foliar product (Dipel®) and the Cry1Ac contained in it [29]. Further selection in the lab using Bollgard II ® foliage which expresses Cry2Ab and Cry1Ac resulted in a Bollgard II®-resistant population that can survive on Bollgard II® (Ping Wang, unpublished). *T. ni* is also a pest of crucifers and can survive and reproduce on Cry1Ac producing broccoli [30].

Having these Bt plants and the insects resistant to the proteins produced in them has allowed us to investigate the effects of these Cry proteins on *C. rufilabris* without the potential confounding effects of prey quality. Prey quality effects can occur when Cry protein-susceptible insects are fed to natural enemies and the reduced quality of the host insects result in reduced growth and development of the natural enemies [7], [8]. Using resistant insects has allowed us to investigate all presently commercially available Bt proteins for control of Lepidoptera: Cry1Ab (maize), Cry1Ac (cotton), Cry2Ab (cotton and maize) and Cry1F (maize). Cry1Ab and Cry1Ac are closely related and share the same binding sites, at least in the European corn borer [31], while each of the other proteins are considered to have distinctly different binding sites [32], [33]. Our use of the Cry1A, Cry2Ab and Cry1F proteins expressed in plants, consumed by resistant Lepidoptera and fed to *C. rufilabris,* provides a unique system to investigate the effects of these proteins on this important predator in cotton and maize agroecosystems. The evidence is clear from our studies that these proteins do not harm *C. rufilabris*, even though they were exposed to these toxins in a bioactive form.

The important role of natural enemies like *C. rufilabris* in Bt crops is two-fold. The more commonly promoted role is that their preservation will help suppress populations of both major and minor pests in the agroecosystem that are not controlled by Bt proteins, such as plant bugs, whiteflies, thrips, aphids and mites. However, a secondary role is that generalists natural enemies may also help suppress the pest population targeted by the Bt proteins produced in the plant. This question was first studied by Gould et al. in their conceptual and mathematical models on tritrophic interactions of a plant, an herbivore and a natural enemy [34]. Recent work in the Shelton laboratory with *P. xylostella*, Bt broccoli and the generalist predator, *Coleomegilla maculata* (Coleoptera: Coccinellidae), demonstrated that this natural enemy can delay the evolution of resistance in *P. xylostella* to the Cry1Ac protein expressed in Bt broccoli (unpublished). These data suggest that natural enemies can play a significant role in insecticide resistance management in Bt crops.

## Materials and Methods

### Plant Materials

Transgenic broccoli (*Brassica oleracea* L., var. ‘*italica*’ ‘Green Comet’), which produces high levels of Cry1Ac, was used in this study [30]. Non-Bt broccoli (Packman F1 Hybrid) (Harris® Seeds, Rochester, NY), a similar variety of broccoli, was used as control since ‘Green Comet’ is no longer available. Plants were grown in 6 L plastic pot in the same greenhouse at 17±2°C under a light and dark regime of 16∶8 h.

Seeds of Bt cotton Bollgard II® (Event 15985), producing Cry1Ac and Cry2Ab, and the corresponding non-transformed near isoline Stoneville 474 were obtained from Monsanto (St. Louis, MO). Bt cotton and non-Bt cotton were grown in 6 L plastic pots in the same greenhouse at 27±2°C under a light and dark regime of 16∶8 h.

Seeds of Bt maize (Mycogen 2A517), carrying the gene coding for Cry1F, and the corresponding non-transformed near isoline (Mycogen 2A496) were obtained from Dow AgroSciences (Indianapolis, IN). Bt maize and non-Bt maize were grown in Ray Leach Cone-tainer Cells (diameter 3.8 cm; depth 21 cm; volume 164 ml) (Stuewe & Sons, Tangent, OR) in the same greenhouse at 21±2°C under a light and dark regime of 16∶8 h.

### Insects

The Bt-susceptible *T. ni* strain was maintained on an artificial diet in the laboratory for >20 years without exposure to Bt toxins [35]. The Cry1Ac-resistant strain (GLEN-Cry1Ac-BCS) was originally collected from commercial greenhouses in British Columbia, Canada and further selected with Cry1Ac and backcrossed with the susceptible laboratory strain. The Cry1Ac/Cry2Ab- resistant strain (GLEN-BGII) also originated from the Bt-resistant greenhouse populations in British Columbia and was selected on Bollgard II® foliage in the laboratory. Previous studies have shown that larvae from the GLEN-Cry1Ac colony can survive well and complete their development on Bt plants expressing Cry1Ac and the GLEN-BGII larvae do likewise on Bollgard II® cotton [14], [35].

A Cry1F-resistant strain of *S. frugiperda* was obtained from Dow AgroScience in 2010 and maintained in our laboratory on artificial diet. This strain developed resistance to Cry1F maize in Puerto Rico and is able to survive on Cry1F maize [15].

A strain of *P. xylostella* susceptible to Cry1Ab, Cry1Ac, Cry1F and Cry2Ab (Bt-susceptible strain),which has been continuously reared on artificial diet since 1988, was used to assess the bioactivity of Bt proteins [36]. Second instars of *P. xylostella* were used in bioassays, as described below.

Eggs of the green lacewing, *C. rufilabris*, were obtained from Beneficial Insectary Inc. (Redding, CA). Tri-trophic bioassays were initiated with newly hatched 1^st^ instar larvae.

All insect strains were maintained in a climatic chamber at 27±1°C, 50±10% RH, and 16∶8 h photoperiod. All experiments were conducted under these conditions as well.

### Prey-mediated Effects of Cry1Ac Broccoli on *C. rufilabris*

First instar *C. rufilabris* were individually kept in 30-ml Cometware ™ plastic cups (WNA, Covington, KY) and supplied with either 1^st^ or 2^nd^ instar Bt-susceptible *T. ni* fed control broccoli, Cry1Ac-resistant *T. ni* fed control broccoli, or Cry1Ac-resistant *T. ni* fed Cry1Ac broccoli. A piece of control broccoli leaf was placed in each cup and a water-saturated cotton ball was provided on the bottom of each cup to maintain humidity. *T. ni* were changed daily and *C. rufilabris* were checked twice daily and the survival, and developmental time of larvae and pupae were recorded. The experiment was initiated with 30 *C. rufilabris* larvae for each treatment.

For assessing fecundity, 8 pairs of newly emerged *C. rufilabris* adults from each treatment were kept in individual transparent plastic cylinders (6.0 cm diameter, 8.5 cm high) and allowed to mate. Each plastic cylinder was covered with a lid, which contained a 4 cm opening to allow ventilation. Between the cylinder and lid, a layer of cotton gauze prevented escape and served as an oviposition substrate. Water was provided by a cotton dental wick, which was positioned through a hole (1cm diameter) at the bottom of each container. The cylinders were placed closely over a water reservoir so that the wicks were submerged and a continuous water supply was ensured. Water in the reservoir was replaced once a week. Adults were fed an artificial diet consisting of sucrose, brewer's yeast and water (in proportions 7∶4:4) for 28 d. Eggs of *C. rufilabris* were removed and recorded daily.

To investigate egg-hatching rates, 30 eggs from each treatment were randomly selected and placed into individual 30-ml cups and monitored until eggs hatched; 3 replications were utilized.

The offspring (F2 of *C. rufilabris*) underwent another generation of testing, as described above.

### Prey-mediated Effects of Cry1Ac/Cry2Ab Cotton on *C.* *rufilabris*

Bioassays were carried out as described above but using the Cry1Ac/Cry2Ab-resistant strain of *T. ni* and Bollgard II® and non-transformed cotton plants.

### Prey-mediated Effects of Cry1F Maize on *C. rufilabris*

First instar *C. rufilabris* were individually kept in 30-ml cups and supplied with either 1^st^ or 2^nd^ instar Cry1F-resistant *S. frugiperda* fed control or Cry1F maize. A piece of control maize leaf was placed in each cup and a water-saturated cotton ball was provided on the bottom of each cup to maintain humidity. *S. frugiperda* were changed daily and *C. rufilabris* were checked twice daily, and the survival, developmental time of larvae and pupae were recorded. The experiment was initiated with 50 *C. rufilabris* larvae for each treatment.

Bioassays for assessing fecundity and egg-hatching rate were conducted as described in the tri-trophic bioassay with Cry1Ac broccoli, *T. ni* and *C. rufilabris*.

The offspring (F2 of *C. rufilabris*) underwent another generation of testing, as described above.

### Bt Protein Residue in Insects

For each bioassay, another 50 1^st^ instar *C. rufilabris* were reared for each treatment as described above. Three samples (6–10 insects as one replicate) from each treatment were collected when *C. rufilabris* reached the 3^rd^ stadium. Three samples of Bt and non-Bt crop leaves (20 mg per replication) and prey (*T. ni* and *S. frugiperda*, 10 larvae per replication) that were used in bioassays were also collected. The Bt protein concentrations in the samples were determined by ELISA using Cry1Ac and Cry2Ab detection kits from EnviroLogix (Portland, ME) and Cry1F detection kits from Agdia (Elkhart, IN). Prior to analysis, all insects were washed with PBST buffer (137 mM NaCl, 2.7 mM KCl, 10 mM Na_2_HPO_4_, 2 mM KH_2_PO_4_, 0.05% Tween-20, pH 7.4) four times to remove any Bt toxin from the surface. Leaf samples were diluted at a rate of 1∶1000 (mg sample: µl PBST buffer) and fully ground with a mortar and pestle. Insect samples were diluted at a rate of 1∶10 (mg sample: µl PBST buffer) in 1.5 ml centrifuge tubes, and ground by hand using a plastic pestle. ELISA was performed according to the manufacturer’s instructions.

### Bioactivity of Bt Proteins after Ingestion by *T. ni* and *S.* *frugiperda*

*T. ni* and *S. frugiperda* used in bioassays were collected and washed with PBST buffer four times and then crushed and diluted at a rate of 1∶20 (mg sample: µl dH_2_O). Bond-spreader sticker (Loveland Industry, Loveland CO) was added at 0.1% to each sample solution before being applied to cabbage leaf disks (diameter 3 cm). Ten 2^nd^ instar Bt-susceptible *P. xylostella* were placed on each of the leaf disks inside 30-ml cups with 5 replicates per treatment. Larval mortality was assessed after 72 h at 27±1°C.

### Statistical Analyses

Data on Bt proteins level in plant tissues and insects were analyzed using one-way analysis of variance (ANOVA) and Tukey’s multiple-range test. Data on survival of *C. rufilabris* were analyzed using the Wilcoxon test for homogeneity. Data on other life table parameters of *T. ni*-fed *C. rufilabris* were subjected to one-way ANOVA and Tukey’s multiple-range test. Data on life table parameters of *S. frugiperda*-fed *C. rufilabris* were analyzed using Student’s *t*-test. Data on bioactivity of Bt proteins were analyzed using one-way ANOVA and Tukey’s multiple-range test. Before analysis, all percentage data were arcsine or square root transformed, as necessary, but untransformed means are presented. All statistical analyses were performed with SAS version 9.1 [37]. For all tests, α = 0.05.
